# 

**Published:** 1905-03

**Authors:** 


MARCH, 1905.

*Vol. XXIII.* No. 87.

INDEXED S. G. O.

### THE BRISTOL MEDICO-CHIRURGICAL JOURNAL

*PUBLISHED UNDER THE AUSPICES OF*
*THE BRISTOL MEDICO-CHIRURGICAL SOCIETY.*

*Editor:* R. SHINGLETON SMITH, M.D., B.Sc.

WITH WHOM ARE ASSOCIATED

J. MICHELL CLARKE, M.A., M.D.,

J. LACY FIRTH, M.S., M.D., JAMES SWAIN, M.S., M.D.

P. WATSON WILLIAMS, M.D., *Assistant Editor.*

*Editorial Secretary:* JAMES TAYLOR.

LIBRARY SURGEON GENERAL'S OFFICE
APR. 4 - 1905

*Bath* ... ... EDWARD J. CAVE, M.D.
*Cardiff* ... ... D. R. PATERSON, M.D., C.M.
*Cheltenham* ... E. T. WILSON, M.B.
*Exeter* ... ... W. GORDON, M.A., M.D.
*Gloucester* ... RAYNER W. BATTEN, M.D.
*Newport* ... A. GARROD THOMAS, M.D., C.M.
*Plymouth* . PAUL SWAIN, F.R.C.S.
*Swansea* ... E. LECRONIER LANCASTER, B.A., M.B., B.Ch.
*Swindon* ... J. CAMPBELL MACLEAN, M.B., C.M.
*Weston-s.-Mare* R. ROXBURGH, M.B.

BRISTOL: J. W. ARROWSMITH,
LONDON: J. & A. CHURCHILL, 7 GREAT MARLBOROUGH STREET.

*Price One Shilling and Sixpence.*

